# Somatotroph Tumors and the Epigenetic Status of the *GNAS* Locus

**DOI:** 10.3390/ijms22147570

**Published:** 2021-07-15

**Authors:** Pauline Romanet, Justine Galluso, Peter Kamenicky, Mirella Hage, Marily Theodoropoulou, Catherine Roche, Thomas Graillon, Heather C. Etchevers, Daniel De Murat, Grégory Mougel, Dominique Figarella-Branger, Henry Dufour, Thomas Cuny, Guillaume Assié, Anne Barlier

**Affiliations:** 1 Aix Marseille Univ, INSERM, APHM, MMG, UMR1251, Marmara Institute, La Conception, Hospital Laboratory of Molecular Biology, 13385 Marseille, France; pauline.romanet@univ-amu.fr (P.R.); galluso_justine@hotmail.fr (J.G.); gregory.mougel@ap-hm.fr (G.M.); 2 Université Paris-Saclay, Inserm, Physiologie et Physiopathologie Endocriniennes, Assistance Publique-Hôpitaux de Paris, Hôpital Bicêtre, Service d’Endocrinologie et des Maladies de la Reproduction, Centre de Référence des Maladies Rares de l’Hypophyse, 94270 Le Kremlin-Bicêtre, Île-de-France, France; peter.kamenicky@aphp.fr (P.K.); mirella.hage@gmail.com (M.H.); 3 Medizinische Klinik und Poliklinik IV, LMU Klinikum, Ludwig Maximilian University Munich, 80336 Munich, Germany; Marily.Theodoropoulou@med.uni-muenchen.de; 4 APHM, La Conception Hospital, Laboratory of Molecular Biology, 13385 Marseille, France; catherine.roche@ap-hm.fr; 5 Aix Marseille Univ, INSERM, APHM, MMG, UMR1251, Marmara Institute, La Timone Hospital Department of Neurosurgery, 13385 Marseille, France; thomas.graillon@ap-hm.fr (T.G.); henry.dufour@ap-hm.fr (H.D.); 6 Aix Marseille Univ, INSERM, MMG, UMR1251, Marmara Institute, 13385 Marseille, France; heather.etchevers@inserm.fr; 7 Université de Paris, Institut Cochin, Inserm U1016, CNRS UMR8104, F-75014 Paris, France; daniel.de-murat@inserm.fr (D.D.M.); guillaume.assie@aphp.fr (G.A.); 8 Aix-Marseille Univ, APHM, CNRS, INP, Inst Neurophysiopathol, CHU Timone, Service d’Anatomie Pathologique et de Neuropathologie, 13385 Marseille, France; DominiqueFrance.FIGARELLA@ap-hm.fr; 9 Aix Marseille Univ, INSERM, APHM, MMG, UMR1251, Marmara Institute, Department of Endocrinology, Hospital La Conception, 13385 Marseille, France; thomas.cuny@ap-hm.fr; 10 Department of Endocrinology, Center for Rare Adrenal Diseases, Assistance Publique—Hôpitaux de Paris, Hôpital Cochin, 75014 Paris, France

**Keywords:** pituitary, *GNAS*, gsp oncogene, somatotroph, tumorigenesis, epigenetic, imprinting, relaxation, PitNET

## Abstract

Forty percent of somatotroph tumors harbor recurrent activating *GNAS* mutations, historically called the *gsp* oncogene. In *gsp*-negative somatotroph tumors, *GNAS* expression itself is highly variable; those with *GNAS* overexpression most resemble phenotypically those carrying the *gsp* oncogene. *GNAS* is monoallelically expressed in the normal pituitary due to methylation-based imprinting. We hypothesize that changes in *GNAS* imprinting of *gsp*-negative tumors affect *GNAS* expression levels and tumorigenesis. We characterized the *GNAS* locus in two independent somatotroph tumor cohorts: one of 23 tumors previously published (PMID: 31883967) and classified by pan-genomic analysis, and a second with 82 tumors. Multi-omics analysis of the first cohort identified a significant difference between *gsp*-negative and *gsp*-positive tumors in the methylation index at the known differentially methylated region (DMR) of the *GNAS* A/B transcript promoter, which was confirmed in the larger series of 82 tumors. *GNAS* allelic expression was analyzed using a polymorphic Fok1 cleavage site in 32 heterozygous *gsp*-negative tumors. *GNAS* expression was significantly reduced in the 14 tumors with relaxed *GNAS* imprinting and biallelic expression, compared to 18 tumors with monoallelic expression. Tumors with relaxed *GNAS* imprinting showed significantly lower *SSTR2* and *AIP* expression levels. Altered A/B DMR methylation was found exclusively in *gsp*-negative somatotroph tumors. 43% of *gsp*-negative tumors showed *GNAS* imprinting relaxation, which correlated with lower *GNAS*, *SSTR2* and *AIP* expression, indicating lower sensitivity to somatostatin analogues and potentially aggressive behavior.

## 1. Introduction

Somatotroph pituitary neuroendocrine tumors (PitNET) are benign tumors of the anterior pituitary gland. Somatotroph PitNET are growth hormone (GH) hypersecreting, leading to overproduction of insulin-like growth factor 1 (IGF-1) and resulting in gigantism in children and acromegaly in adults. Surgical removal leads to normalization of GH and IGF1 in 34–74% of cases depending on the series, related mostly to tumor size [1]. GH synthesis and secretion is primarily under stimulatory control of GH-releasing hormone (GHRH), while somatostatin inhibits GH release, mainly through the subtype 2 of the somatostatin receptor (SSTR2) [2]. SSTR2 is the primary target of the somatostatin analogues (SSAs) currently used to treat these tumors. However, 50% of patients remain resistant or partially resistant to these drugs due to a loss of SSTR2 expression [3].

Approximately 40% of somatotroph PitNET harbor activating mutations in the *GNAS* gene (NM_000516.7; HGNC:4392) encoding the α-subunit of the heterotrimeric GTP-binding protein (G_s_α) [4,5]. Products of *GNAS* mutated at arginine 201 (R201) or glutamine 227 (Q227) are historically known as the *gsp* oncogene [4]. We have previously shown that the *gsp* oncogene impacts the tumoral phenotype, with *gsp*-positive tumors being smaller and more sensitive to treatment with the SSTR2 agonist octreotide, than *gsp*-negative ones [6,7,8,9,10].

**Figure 1:**
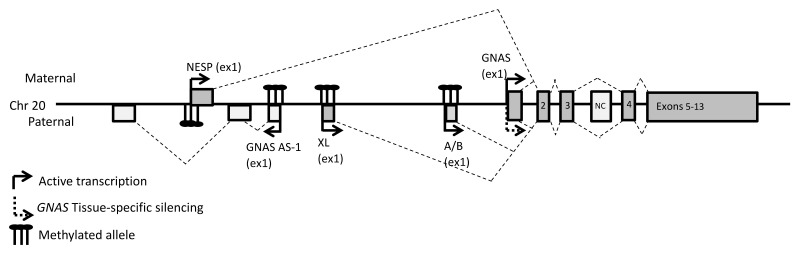
General organization of the *GNAS* locus. The dark gray and light gray boxes represent the coding and noncoding exons, respectively. The broken lines indicate the splicing patterns. Numbers indicate the numbers of the exons.

The mRNA encoding for G_s_α is one of the multiple transcripts from the complex imprinted *GNAS* locus on chromosome 20q11 [11] (Figure 1). Four distinct sense transcripts, three of which encode one or more peptides, are generated by alternative promoters and first exons that splice onto the second of twelve additional 3′ exons. However, there is evidence of additional transcript variants, some of which translate to distinct protein isoforms (Supplemental Table S1). The first exon of the *NESP* transcript (NM_016592.5) is the furthest 5′ relative to the first exon of *GNAS*, at 49 kilobases (kb) upstream. *NESP* encodes the 55 kDa neuroendocrine secretory protein (NESP55 or SCG6), a chromogranin-like protein. Thirty-five kb upstream of *GNAS* exon 1 is the first exon of the *XL* mRNA (NM_080425.4). *XL* encodes “extra-large alpha-s” (XLas), an isoform of *GNAS* with a long amino-terminal extension. This transcript also contains an alternative reading frame that can be translated into the unrelated protein ALEX (alternative gene product encoded by XL-exon), which interacts with XLas to restrain its adenylate cyclase stimulatory activity [12]. About 2.5 kb upstream of *GNAS*, a fourth alternative first exon defines the start of the noncoding *A/B* transcript (NR_132273.1) a long non-coding (lnc)RNA lacking a translation initiation codon that is also transcribed as a shorter splice variant (NR_132272.2). Finally, *GNAS-AS1* (NR_002785.2) is another lncRNA, whose first antisense facing exon is found between the first exons of *NESP* and *XL* (Figure 1).

The *GNAS* locus is imprinted at several differentially methylated regions (DMR) in the promoters of all *GNAS* locus transcripts, except *GNAS* itself (Figure 1). *NESP* is expressed exclusively from the maternal allele, due to imprinted methylation of its paternal DMR. *GNAS-AS1*, *XL* and *A/B* are expressed from the paternal allele, due to methylation of their respective DMRs on the maternal allele. *GNAS* is biallelically expressed in all tissues except a few, including the pituitary, the renal proximal tubule, the thyroid and the gonads [8,13,14]. In these tissues, *GNAS* is exclusively expressed from the maternal allele, and the imprinting that affects *GNAS* transcription occurs through the differential methylation of A/B upstream of the *GNAS* promoter in both animal models and humans [15]. It is suspected that tissue-specific maternal methylation of the A/B DMR normally prevents binding of an unidentified to date *GNAS* transcriptional repressor. This mechanism would explain pseudohypoparathyroidism type 1B, a rare disease characterized by DMR hypomethylation at A/B with concomitant loss of *GNAS* expression in tissues where *GNAS* is normally imprinted [16].

We have shown that in *gsp*-positive somatotroph PitNETs, the mutation occurs almost exclusively on the maternal allele, reflecting *GNAS* imprinting [8]. *GNAS* mRNA levels are highly variable among somatotroph PitNETs. The highest expression is observed in *gsp*-negative tumors [9,17,18] and is associated with smaller tumor size and higher sensitivity to SSA [19]. *GNAS* imprinting relaxation is observed in some somatotroph PitNETs [8,19], but the mechanism underlying this relaxation has not been elucidated.

We hypothesize that epigenetic defects on the *GNAS* locus are involved in the relaxation of *GNAS* imprinting and *GNAS* expression and could consequently contribute to somatotroph tumorigenesis.

## 2. Materials and Methods

### 2.1. Somatotroph Tumors

This work included 2 independent cohorts of somatotroph tumors selected on histopathological criteria: cohort I included 23 somatotroph tumors from a previously published large collection of 134 PitNETs that was used to support a pan-genomic classification of PitNETs [20]. Genomic, transcriptomic and epigenetic data were reanalyzed as previously described [20], focusing on the *GNAS* locus. Cohort II included 82 additional somatotroph tumors, fifteen of which treated by SSAs before surgery. In addition, six normal human pituitaries serve as controls for the cohort II.

### 2.2. Genetic and Epigenetic Analysis

Genomic DNA of cohort II was extracted using the QIAamp DNA Mini kit (Qiagen, Courtabœuf, France). *Gsp*-oncogene was identified by Sanger sequencing targeting exons 8 and 9 of *GNAS* (*GNAS* exon 8-F TGAGCCTGACCTTGTAGAGAGACAC, *GNAS* exon 8-R AACATGCTGGTGGGGAGGAGGACAG, *GNAS* exon 9-F TCATGGTTTCTTGACATTCACC, *GNAS* exon 9-R TAA ACA GTGCAGACCAGGGC) on an AB3130XL (ThermoFisher Scientific, Waltham, MA, USA).

The methylation level of the *A/B* DMR (A/B ML) was determined by pyrosequencing on a Pyromark MD (Qiagen) after bisulfite conversion using the Epitect Bisulfite Kit (Qiagen) with the Hs_GNAS_04_PM_Q96 methylation assay kit (Qiagen) [21]. This assay targets 6 CpG doublets at the position chr20(GRCH37):57463600-57463700. The A/B ML was calculated from the mean of the methylation at the 6 CpG doublets. Three of these 6 CpG doublets correspond to the cg26767990, cg17652507, and cg22407822 of the Infinium^®^ MethylationEPIC BeadChip (Illumina) used in the pan-genomic study [20]. These 3 CpG doublets were used to determine the methylation indices of A/B DMR (A/B MI) in the DNA methylation profiling of cohort I.

### 2.3. Expression Analysis of GNAS, SSTR2 and AIP of Cohort II:

For RNA extraction, 30 mg of each pituitary fragment conserved at −80 °C was ground over dry ice using a FastPrep device (MP Biomedical, Illkirch Cedex, France). The total RNA fraction was extracted with the RNeasy Plus Mini kit (Qiagen). Reverse transcription was performed with the First Strand cDNA Synthesis kit (GE Healthcare, Tremblay-en-France, France) according to the manufacturer’s instructions. The β-glucuronidase (*BGUS*) and *GNAS* transcripts were quantified using in-house assays as previously described [19]. The *AIP* and *SSTR2* transcripts were quantified using commercial assays hSST2 (ID Hs00265624_s1) and hAIP (ID Hs00610222_m1, Applied Biosystems) respectively. Quantitative PCR was performed with the TaqMan gene expression kit (Applied Biosystems, Waltham, MA, USA) on a Viia7 thermal cycler (Roche Diagnostics, Meylan, France). A plasmid range was performed for each assay and absolute quantification was performed, normalized to the amount of *BGUS* housekeeping transcripts for each sample.

### 2.4. Allelic Quantification (AQ) of GNAS Expression

The AQ of *GNAS* expression (quantification of allele-specific *GNAS* expression) was based on the polymorphism that induces sensitivity to the Fok1 restriction endonuclease in *GNAS* exon 5, rs7121, c.393C > T, p.(Ile131Ile), which is expressed in the general population at an allelic frequency of 35%. Only somatotroph tumors and normal pituitary glands that were heterozygous for the Fok1 polymorphism were selected. *GNAS* AQ was done by pyrosequencing, using one pair of PCR primers, the forward of which is specifically located in exon 1 of *GNAS*, and the reverse of which carries a biotin group, targeting sequence includes the Fok1 position, and a sequencing primer, adjacent to the Fok1 position (RT-PCR-primer-F CGTGAGGCCAACAAAAAGAT, RT-PCR-primer-R: biotin-ATGGCAGTCACATCGTTGAA, pyroseq-primer F: GAGAACCAGTTCAGAGT) [22]. Mono-allelic *GNAS* expression was distinguished from a bi-allelic one (Supplemental Figure S1) using a threshold value determined from normal pituitaries, expressing *GNAS* only from the maternal allele.

### 2.5. Statistical Analysis

Statistical analyses were performed using Prism v9.0 (GraphPad Software, La Jolla, CA, USA). The Agostino and Pearson normality test was used to determine if methylation and expression values followed a normal distribution or not. This test computes skewness and kurtosis to quantify how far the distribution is from Gaussian. It then calculates how far each of these values differs from the value expected in a Gaussian distribution. If the distribution is normal, the means ± 2SD were considered to be within the threshold of “normal” values for *A/B* methylation and *GNAS* expression. Tumor characteristics were compared using the unpaired non-parametric Mann–Whitney test for quantitative values, and the Fisher exact test for qualitative values.

## 3. Results

### 3.1. Cohort I

Twenty-three somatotroph tumors were selected from a large cohort of 134 pitNETs classified in 6 categories (t1 to t6) according to their transcriptomic profiles obtained by RNAseq [20]. Six somatotroph tumors (6/23, 26% of somatotroph tumors) clustered in the t5 class, which also included five thyrotroph and five pluri-hormonal Pit1-positive PitNETs. The 17 others were assigned to the t6 class named the “somatotroph group”, which was subdivided into two subgroups, t6a and t6b. The t6a group included seven somatotroph tumors (7/23, 30% of the somatotroph tumors) and two mixed somato-lactotroph tumors. The t6b group included 10 somatotroph tumors (10/23, 44% of somatotroph tumors), six mixed somato-lactotroph tumors and two pluri-hormonal Pit1-positive tumors. The mixed somato-lactotroph tumors and the pluri-hormonal Pit-1 tumors were excluded from this analysis.

**Figure 2:**
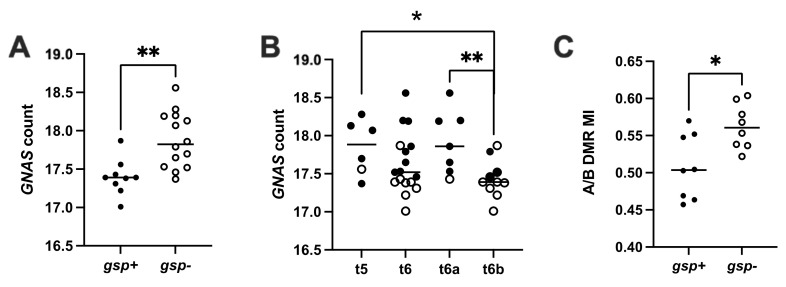
*GNAS* expression and methylation indices of A/B DMR determined by pan-genomic analysis of the somatotroph tumors from the cohort I. *GNAS* count by TPM estimated from transcriptomic analysis using DESeq2. A/B DMR MI: methylation indices of A/B DMR. (**A**) *gsp*-negative tumors show higher *GNAS* transcription than *gsp*-positive tumors. (**B**) Differential *GNAS* expression in tumors after pan-genomic classification. Tumors from the t5 and t6a subgroups express more *GNAS* than t6b tumors. (**C**) Differential methylation indices (MI) at the A/B DMR in *gsp*-negative and *gsp*-positive tumors of cohort I. *: *p* < 0.05; **: *p* < 0.01; *gsp*-: tumors without the *gsp* oncogene, white dots; *gsp*+: tumors with the *gsp* oncogene, black dots.

Sequencing revealed 14 *gsp*-negative (61%, 14/23) and nine *gsp*-positive (39%, 9/23) tumors. Among the *gsp*-positive tumors, 7/9 were assigned to the t6b subgroup, one to t5, and the last to t6a. The *GNAS* mRNA levels obtained by transcriptomic analysis (normalized counts of transcripts per million as calculated with DESeq2) were lower in *gsp*-positive tumors than in *gsp*-negative ones (median 17.4 counts vs. 17.8, *p* = 0.002, Figure 2A). Accordingly, the t6b subgroup had significantly lower *GNAS* expression than t6a tumors (t6a median = 17.9, t6b median = 17.4, *p* = 0.009). The gene copy number analysis was available for 15 somatotroph tumors. A copy gain on chromosome 20q was observed for three, including one *gsp*-positive tumor, but *GNAS* expression was not significantly increased in comparison to tumors without copy gain. The A/B DMR methylation indices (A/B MI) were significantly higher in *gsp*-negative tumors compared to *gsp*-positive tumors (median 0.56 vs. 0.50 respectively, *p* = 0.028, Figure 2C).

### 3.2. Cohort II

#### 3.2.1. *Gsp* Oncogene Status and *GNAS* Expression

Nineteen somatotroph tumors carried *gsp*. The *GNAS*, *AIP*, and *SSTR2* mRNA expression levels were not statistically different between presurgical SSAs treated and un-pretreated *gsp*-negative tumors.

**Figure 3:**
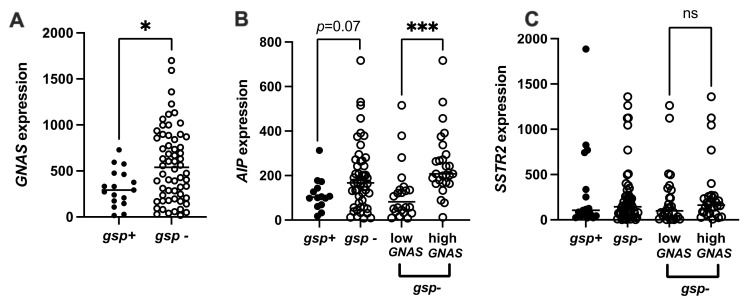
Characterization of *GNAS*, *AIP* and *SSTR2* expression in the cohort II. (**A**) *GNAS* expression (copies/copy × 1000 *BGUS*) in *gsp*-negative tumors versus *gsp*-positive tumors. (**B**) *AIP* expression (copies/copy × 1000 *BGUS*) in *gsp*-negative tumors versus *gsp*-positive tumors, and in *gsp*-negative tumors according to *GNAS* expression levels. (**C**) *SSTR2* expression (copies/copy × 1000 *BGUS*) in *gsp*-negative tumors versus *gsp*-positive tumors and in *gsp*-negative tumors according to *GNAS* expression levels. *: *p* < 0.05; ***: *p* < 0.001; *gsp*-: tumors without the *gsp* oncogene, white dots; *gsp*+: tumors with the *gsp* oncogene, black dots. Low *GNAS* or high *GNAS*: *gsp*-negative tumors expressing respectively greater or fewer than 517 copies per copy of *BGUS*, × 1000. *: *p* < 0.05. ***: *p* < 0.001.

As in cohort I, the *GNAS* mRNA levels in the 19 *gsp*-positive tumors were lower than in the 63 gsp-negative ones (median 293 vs. 536 × 1000 copies per copy *BGUS p* = 0.019, Figure 3A). The *AIP* mRNA expression level was also lower, but this was not significant (median 102.7 vs. 167.2 × 1000 copies/copy *BGUS*, *p* = 0.07, Figure 3B). In contrast, *SSTR2* expression levels were similar in both groups (median 106 vs. 144 × 1000 copies/copy *BGUS p* = 0.9, Figure 3C). *GNAS* mRNA expression levels followed a normal distribution in *gsp*-positive tumors (*p* = 0.7), but not in *gsp*-negative tumors. Therefore, we used the values of *GNAS* expression in *gsp*-positive tumors to determine an overexpression threshold for *GNAS*. In *gsp*-negative tumors, *GNAS* was considered to be overexpressed when the value was higher than the mean ± SD (517 × 1000 copies/copy *BGUS*). Among the 63 *gsp*-negative tumors of cohort II, 34 overexpressed *GNAS*. In this subgroup, the *AIP* expression level was also significantly higher (median 82.4 vs. 20.7 × 1000 copies/copy *BGUS p* = 0.0001, Figure 3B), while the *SSTR2* expression level remained not statistically different (median 99 vs. 167 × 1000 copies/copy *BGUS*, *p* = 0.2; Figure 3C).

#### 3.2.2. Methylation Levels of A/B DMR (A/B ML)

**Figure 4:**
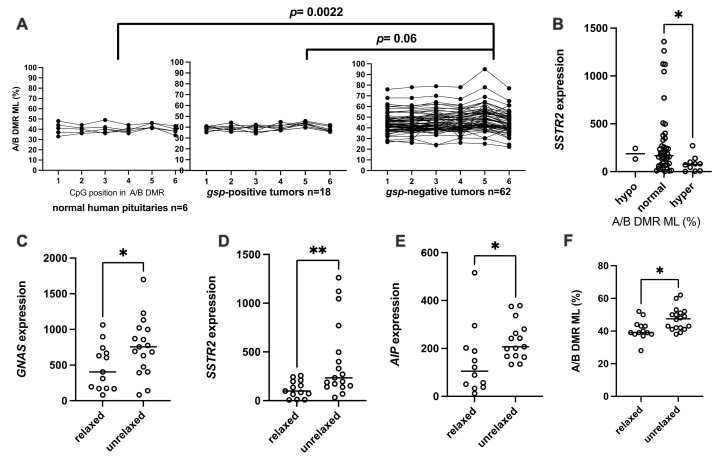
Characterization of *GNAS*, *AIP* and *SSTR2* expression in the “82 tumors” cohort according to the epigenetic disturbance of *GNAS* locus. A/B DMR ML: methylation level of A/B DMR. (**A**) pattern of A/B DMR methylation in normal human pituitary gland, *gsp*-positive somatotroph adenomas and *gsp*-negative somatotrophs adenomas. (**B**) *SSTR2* expression (copies/copy × 1000 *BGUS*) in *gsp*-negative tumors as a function of *A/B* DMR methylation levels. (**C**) *GNAS*, (**D**) *SSTR2*, (**E**) *AIP,* mRNA expression (copies/copy × 1000 *BGUS*) in *gsp*-negative tumors according to the relaxation of *GNAS* imprinting. (**F**) Methylation level of A/B DMR in relaxed and unrelaxed *gsp*-negative tumors. *: *p* < 0.05; **: *p* < 0.01.

The A/B ML median was at 39% (range 36–46%) in the normal pituitaries and covered the same range in the *gsp*-positive tumors (median 40%, 37–42%, Figure 4A). In contrast, A/B ML were highly variable in the *gsp*-negative adenomas (median 43%, 25–81%), and different from that of normal pituitaries (*p* = 0.022) or *gsp*-positive tumors (*p* = 0.06). The A/B ML in *gsp*-positive tumors followed a normal distribution (*p* = 0.98). The mean ± 2SD in this group was chosen as the threshold to assign a hypo- or hyper-methylated status (42.4 ± 11.4%). Two *gsp*-negative tumors (3%) were thus hypo-methylated at the A/B DMR while 12 (20%) were A/B-hyper-methylated. Somewhat surprisingly, they also showed no correlation to the *GNAS* expression level. *GNAS* expression was lower, but not significantly so, in these *A/B*-hyper-methylated *gsp*-negative tumors relative to the normo-methylated ones (median 378 vs. 603 × 1000 copies/copy *BGUS*, *p* = 0.18). *AIP* expression levels showed a similar tendency (median 150.8 vs. 179.5 × 1000 copies/copy *BGUS*, *p* = 0.5). Only *SSTR2* expression levels were significantly lower in the A/B-hyper-methylated, *gsp*-negative tumors compared to the normo-methylated ones (median 76 vs. 167 × 1000 copies/copy *BGUS, p* = 0.012, Figure 4B).

#### 3.2.3. Relaxation of *GNAS* Imprinting

Thirty-two *gsp*-negative tumors and 3 normal pituitary fragments were heterozygous for a known SNV that induces a Fok1 restriction enzyme cleavage site, and thereby informative for allelic quantification (AQ). In the normal pituitary, the *A/B* DMR upstream of *GNAS* is paternally imprinted, such that only the maternal allele of *GNAS* is expressed in this tissue. Using the AQ of normal pituitaries as a reference, “relaxation” of *GNAS* imprinting was defined as when *GNAS* expression from the minor expressed allele was greater than 20% of total *GNAS* expression. Among the 32 informative *gsp*-negative tumors, 18 were considered normally imprinted while 14 were relaxed. These 18 unrelaxed, *gsp*-negative tumors transcribed significantly more *GNAS, AIP* and *SSTR2* than the relaxed ones (median 755 vs. 405 × 1000 *GNAS* copies/copy *BGUS*, *p* = 0.05; 634 vs. 203 × 1000 *SSTR2* copies/copy *BGUS*, *p* = 0.02; and 207.5 vs. 105.1 × 1000 *AIP* copies/copy *BGUS*, *p* = 0.007; Figure 4C–E respectively). On the other hand, A/B ML was higher in unrelaxed *gsp*-negative tumors compared to relaxed *gsp*-negative tumors (47.5%, min 38%, max 62%, versus 39%, min 28%, max 52%, *p* = 0.013 Figure 4F).

## 4. Discussion

We hypothesized that epigenetic defects at the *GNAS* locus could impact *GNAS* imprinting, *GNAS* transcription, and ultimately, somatotroph tumorigenesis. This hypothesis was based on the molecular mechanism underlined in pseudohypoparathyroidism type 1B (PHP1B), an imprinting disorder. PHP is a group of rare diseases characterized by end-organ resistance to parathyroid hormone (PTH) but also to GHRH or TSH. The corresponding hormonal receptors are transmembrane receptors coupled to the G_α_s subunit. In some tissues, including the pituitary gland [8], *GNAS* is mono-allelically expressed from the maternal allele. PHP1B develops due to the loss of *GNAS* expression in a tissue where *GNAS* is normally imprinted, following hypo-methylation of the A/B DMR [16]. We therefore investigated whether somatotroph tumorigenesis may have an analogous pathogenetic mechanism to PHP1B, through aberrant methylation at the *GNAS* locus

The *gsp*-oncogene was detected in 39% of somatotroph tumors from the cohort I, and 25% from the cohort II, which findings are consistent with the literature [23]. In the cohort I, the *gsp*-positive tumors mainly clustered in the same transcriptomic class (t6) whereas *gsp*-negative tumors clustered either in t6 or in another class (t5). The pan-genomic classification thus highlights a population of somatotroph tumors that are *gsp*-negative but behave functionally like *gsp*-positive tumors. These tumors, belonging to the t6a subgroup, had higher *GNAS* expression than t6b ones (*p* = 0.009), including the mainly *gsp*-positive tumors.

Transcriptomic and targeted RT-PCR analysis of these independent cohorts I and II confirmed a significant difference of *GNAS* expression level between *gsp*-positive tumors and *gsp*-negative ones, as previously reported [18]. Among the *gsp*-negative tumors, we identified a population over-expressing *GNAS*. This group also expressed higher *AIP* mRNA levels. AIP is a chaperone protein that behaves like a tumor suppressor in somatotroph tumors. High levels of *AIP* mRNA are a marker of reduced invasiveness of somatotroph tumors [24]. AIP-dependent regulation of the cAMP pathway seems to play a predominant role in this protective function [25,26]. Immunohistochemistry has shown that high AIP expression is correlated with better SSA response [27]. Nevertheless, in the literature, *AIP* and *SSTR2* are independent, uncorrelated markers of response to SSAs.

We have demonstrated here for the first time, through methylome analysis and pyrosequencing, differential methylation at the A/B DMR between *gsp*-negative and *gsp*-positive tumors. In the cohort II, we showed that A/B DMR methylation levels were similar in the *gsp*-positive group and in normal pituitaries, while they were highly variable in the *gsp*-negative tumors. As predicted by our hypothesis, hyper-methylation was detected in 20% of *gsp*-negative tumors, in agreement with the higher A/B MI observed in the *gsp*-negative group of cohort I. However, in these, *GNAS* mRNA expression levels were not correlated with the *A/B* ML, perhaps due to the complex epigenetic regulation at the *GNAS* locus.

Relaxation of *GNAS* imprinting was observed in almost half of the 32 informative *gsp*-negative tumors (14/32). Surprisingly, a higher A/B ML was not observed in the relaxed, *gsp*-negative group compared to the unrelaxed, *gsp*-negative one (Figure 4F). On the contrary, A/B ML was higher in the unrelaxed group. This is not in agreement with our first hypothesis, which stated that hypermethylation of A/B DMR would be involved in the increased expression of *GNAS* in some *gsp*-negative tumors by an enhancer mechanism involving a protein binding to the methylated regions. Nevertheless, using KO mice harboring deletion of A/B DMR, Williamson et al. showed that a paternal deletion of this DMR abolishes tissue-specific imprinting of *Gnas* [28]. This result was confirmed by Liu et al. [29]. Their conclusion is that the unmethylated A/B DMR contains a silencer for *Gnas* expression, which is active on the unmethylated paternal allele. However, contrary to what we observed in human tumors, in these models imprinting relaxation resulted in overexpression of *Gnas*. In our previous work, we have shown that in somatotroph tumors the re-expression of the *GNAS* paternal allele is compensated by a decrease in the expression of the *GNAS* maternal allele [19]. Here, the expression of *GNAS* was found higher in unrelaxed tumors (with higher A/B ML) than in relaxed ones (with lower A/B ML, Figure 4C). In unrelaxed, *gsp*-negative tumors, higher expression of *GNAS* was associated with higher levels of *SSTR2* and *AIP* gene expression.

In conclusion, somatic *GNAS* imprinting relaxation and A/B DMR methylation levels have little effect on *GNAS* expression itself in the context of somatotroph tumors, in contrast to what is observed at the germline level in pseudohypoparathyroidism [19]. Overall, our results suggested that the *GNAS* relaxation and loss of A/B DMR methylation could be one of the steps in the dedifferentiation drift including a loss of *GNAS, AIP* and *SSTR2* expression that may contribute to the tumor promotion. Data from the cohort II allows us to distinguish three groups of tumors: *gsp*-positive tumors with low *GNAS* mRNA expression, unrelaxed *gsp*-negative tumors with high *GNAS*, *SST2* and *AIP* expression levels, and relaxed *gsp*-negative tumors with low *GNAS*, *SSTR2* and *AIP* expression levels (Table 1). The relationship between imprinting loss and tumor aggressiveness remains to be clinically determined.

## Figures and Tables

**Table 1:** Characteristics of three groups of somatotroph tumors within cohort II.

	Group 1 *n* = 16	Group 2 *n* = 18	Group 3 *n* = 14
*GNAS* mutational and imprinting status	*gsp*-positive	*gsp*-negative, unrelaxed	*gsp*-negative, relaxed
*GNAS* expression	Low * (*p* = 0.02)	High ** (*p* = 0.05)	Low *
*AIP* expression	Low * (ns)	High ** (*p* = 0.02)	Low **
*SSTR2* expression	Low * (ns)	High ** (*p* = 0.007)	Low **

* Versus expression in *gsp*-negative tumors, ** comparative expression between relaxed and unrelaxed gsp-negative tumors (group 2 and 3).

## Data Availability

MiRNA and mRNA read counts are available at the European Bioinformatics Institute (EMBL-EBI) Array Express under accession numbers E-MTAB-7969 and E-MTAB-7768 respectively. Methylome data are available under EMBL-EBI Array Express accession number E-MTAB-7762. Sequencing data (exome, mRNA and MiRNA sequencing) has been deposited at the European Genome- phenome Archive (EGA) which is hosted at the EBI and the CRG, under accession number EGAS00001003642.

## Supplementary Materials

The following are available online at https://www.mdpi.com/article/10.3390/ijms22147570/s1, Figure S1: Allelic quantification of *GNAS* expression by pyrosequencing, Table S1: No tittle, Table S2: Clincobiological characteristics of patients and tumors from cohort I, Table S3: Biological characteristics of the 82 tumors from cohort II.
