# Supplementary material for: Somatotroph Tumors and the Epigenetic Status of the GNAS Locus
Source: Int J Mol Sci. 2021 Jul 15;22(14):7570. doi: 10.3390/ijms22147570 (PMC8306130; doi:10.3390/ijms22147570)
Supplement: Supplementary file 1 [file ijms-22-07570-s001.zip › ijms-1267093-supplementary.pdf]

Supplemental Table S1.

Transcript (NCBI RefSeq)	Length (nt)	Transcript variant name	Ensembl ID	Strand	Protein (NCBI RefSeq)	Length (aa)	Protein name	Isoform (NCBI)	Notes
NM_000516.7	1854	1 (GNAS)	ENST00000371085.8	plus	NP_000507.1	394	GNAS	GNAS	canonical $G_{\alpha}s$
NM_080425.4	4027	2 (XL)	ENST00000371100.9	plus	NP_536350.2	1037	GNAS	XLas	
NM_080426.4	1809	3 (GNASS)	ENST00000371095.7	plus	NP_536351.1	380	GNAS	GNASS	Lacks an internal exon and uses an alternate splice site, compared to variant 1. This isoform GNASS, also known as alpha-S1, differs in an internal region but has identical N- and C-termini, compared to GNASL.
NM_001077489.4	1866	7	ENST00000265620.11	plus	NP_001070957.1	379	GNAS	g	Missing last codon re: variant GNAS-S
NM_001077488.5	1857	6 (GNASL)	ENST00000354359.12	plus	NP_001070956.1	395	GNAS	f (GNASL)	
NM_016592.5	2551	4 (NESP)	ENST00000371075.7	plus	NP_057676.1	245	NESP/SCG6	NESP/SCG6	neuroendocrine secretory protein / secretogranin VI
NR_132273.1	1075	14 (A/B variant 1)	ENST00000464960.5	plus					
NR_132272.2	716	13 (A/B variant 2)	ENST00000490374.6	plus					differs at 5' and truncated 3' ends
NR_002785.2	1158	GNAS-AS1	ENST00000424094.6	minus				GNAS antisense RNA 1	3 other potential splice isoforms (Ensembl)
NM_001077490.3	4027	2 (ALEX)	ENST00000371100.9	plus	NP_001070958.1	626	ALEX	ALEX	
	2073	GNAS-203 (ALEX)	ENST00000306120.4	plus			ALEX		present in Ensembl/Uniprot
NM_001309840.2	1580	9	ENST00000477931.5	plus	NP_001296769.1	335	ALEX	h	
NM_001309842.2	1114	10	ENST00000371081.5	plus	NP_001296771.1	87	ALEX	i	
NM_001309861.2	1662	11		plus	NP_001296790.1	335	ALEX	h	
NM_001309883.1	3180	12	ENST00000604005.6	plus	NP_001296812.1	626	ALEX	ALEX	

XM_017027812.2	3752	X1 (predicted)	plus	XP_016883301.1	1038	GNAS	X1
XM_017027813.2	3707	X2 (predicted)	plus	XP_016883302.1	1023	GNAS	X2
XM_017027814.2	3704	X3 (predicted)	plus	XP_016883303.1	1022	GNAS	X3
XM_024451872.1	2573	X4 (predicted)	plus	XP_024307640.1	362	GNAS	X4
XM_017027815.1	1455	X5 (predicted)	plus	XP_016883304.1	347	GNAS	X5
XM_017027821.1	1709	X14 (predicted)	plus	XP_016883310.1	245	NESP/SCG6	X9
XM_017027822.1	1382	X15 (predicted)	plus	XP_016883311.1	245	NESP/SCG6	X9
XM_024451873.1	1492	X6 (predicted)	plus	XP_024307641.1	335	ALEX	X6
XM_024451874.1	1578	X7 (predicted)	plus	XP_024307642.1	335	ALEX	X6
XM_024451875.1	1556	X8 (predicted)	plus	XP_024307643.1	335	ALEX	X6
XM_017027816.1	2260	X9 (predicted)	plus	XP_016883305.1	320	ALEX	X7
XM_017027820.1	1446	X13 (predicted)	plus	XP_016883309.1	320	ALEX	X7
XM_017027817.1	1542	X10 (predicted)	plus	XP_016883306.1	320	ALEX	X8
XM_017027818.2	1567	X11 (predicted)	plus	XP_016883307.1	320	ALEX	X8
XM_017027819.1	1506	X12 (predicted)	plus	XP_016883308.1	320	ALEX	X8
XR_002958471.1	2255	X16 (predicted)	plus				

Supplemental Figure S1: Allelic quantification of *GNAS* expression by pyrosequencing

Pyrogram of the quantification of the *GNAS* allelic expression of 2 heterozygous somatotroph adenomas for the Fok1 polymorphism.

The pyrosequencer performs real-time sequencing of the target sequence (AG/AATGTA) by neosynthesis of the complementary strand by injecting the nucleotides in a pre-programmed order (CAGACTGTA). At the position of the Fok1 polymorphism, the device sequentially injects a G then an A. The peak height ratio gives the percentage of strands having incorporated a G or an A.

NB: the nucleotide following the Fok1 polymorphism is an A, hence the peak height of A.

A: Tumor 58: only one allele is expressed (bearing the G).

B: Tumor 29: this tumor shows biallelic expression of *GNAS*.

C: Tumor 82: this tumor shows bi-allelic expression of *GNAS*.

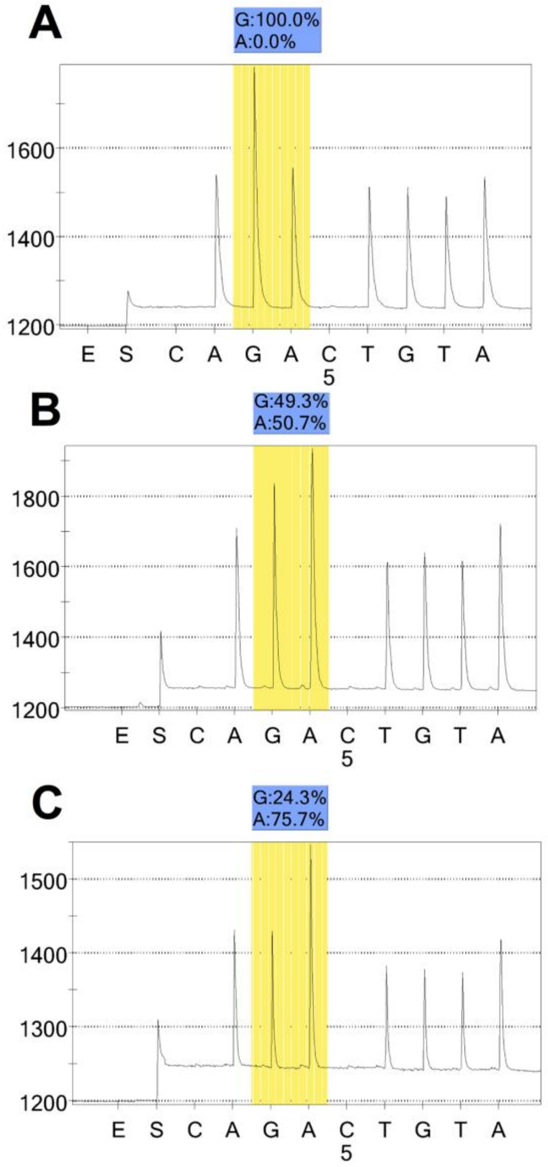

Supplemental Table S2. Clincobiological characteristics of patients and tumors from cohort I N: no, Y: yes, MI: methylation index

Tumor number	age (years)	sexe	grp_tran-scriptome	gsp mutational status	WHO 2017 Tumor Type	*GNAS* expression	cg2676-7990	cg1765-2507	cg2240-7822	Mean MI
P001	38	male	t5	N	Thyreotroph	18,97	0,48	0,47	0,52	0,49
P007	51	female	t5	N	Somatotroph	18,13	0,56	0,50	0,54	0,54
P008	71	male	t5	N	Somatotroph	18,28				
P011	49	female	t5	N	Somatotroph	18,07	0,56	0,49	0,56	0,54
P014	65	female	t5	N	Thyreotroph	17,68	0,53	0,47	0,57	0,52
P015	62	female	t5	N	Plurihormonal PIT-1	18,44	0,47	0,57	0,59	0,54
P018	51	female	t6A	N	Mixed_GH-PRL	16,93	0,53	0,52	0,57	0,54
P019	53	female	t6A	N	Mixed_GH-PRL	18,38	0,60	0,59	0,63	0,60
P020	58	female	t6B	Y	Somatotroph	17,39	0,54	0,53	0,58	0,55
P023	68	female	t5	N	Thyreotroph	18,12	0,50	0,30	0,36	0,39
P024	32	male	t6B	N	Somatotroph	17,52	0,57	0,56	0,57	0,57
P028	29	male	t6B	Y	Somatotroph	17,87	0,49	0,39	0,49	0,46
P031	64	female	t6B	N	Plurihormonal_PIT-1	18,16	0,50	0,53	0,52	0,52
P035	34	male	t6B	Y	Somatotroph	17,01	0,50	0,46	0,55	0,50
P036	49	female	t6B	Y	Mixed_GH-PRL	17,45	0,57	0,57	0,61	0,58
P039	46	female	t6B	Y	Somatotroph	17,39	0,56	0,50	0,59	0,55
P040	26	male	t5	Y	Somatotroph	17,56	0,54	0,47	0,50	0,50
P047	47	female	t5	N	Thyreotroph	19,59	0,56	0,56	0,64	0,59
P051	47	female	t6B	N	Mixed_GH-PRL	18,65	0,62	0,60	0,67	0,63
P057	64	male	t5	N	Plurihormonal_PIT-1	18,12	0,49	0,45	0,45	0,46
P058	65	male	t5	N	Plurihormonal_PIT-1	20,56	0,58	0,55	0,59	0,58
P059	49	male	t6B	Y	Mixed_GH-PRL	16,80				
P060	34	female	t6B	N	Plurihormonal_PIT-1	17,30	0,59	0,60	0,61	0,60
P065	48	male	t6A	N	Somatotroph	18,56				
P067	68	male	t6B	Y	Somatotroph	17,38	0,57	0,52	0,62	0,57
P072	46	male	t6B	N	Somatotroph	17,79	0,60	0,57	0,62	0,60
P076	49	female	t6B	Y	Mixed_GH-PRL	17,28				
P077	30	male	t5	N	Thyreotroph	18,43	0,47	0,37	0,39	0,41
P085	48	female	t5	N	Somatotroph	17,37				
P086	60	male	t6B	Y	Somatotroph	17,22				
P089	37	male	t6A	N	Somatotroph	17,53	0,54	0,50	0,52	0,52
P092	47	female	t5	N	Plurihormonal_PIT-1	18,05				
P093	35	male	t6B	Y	Mixed_GH-PRL	16,95	0,58	0,62	0,66	0,62
P097	49	male	t6B	Y	Somatotroph	17,31	0,32	0,52	0,55	0,46

P099	46	female	t6B	Y	Mixed_GH-PRL	16,47				
P102	53	male	t6B	N	Somatotroph	17,46	0,58	0,54	0,61	0,58
P103	84	male	t6A	N	Somatotroph	18,19	0,59	0,59	0,63	0,60
P107	40	female	t6A	Y	Somatotroph	17,43	0,47	0,43	0,50	0,47
P119	64	male	t6A	N	Somatotroph	17,65				
P123	41	male	t6A	N	Somatotroph	18,20				
P129	22	female	t5	N	Somatotroph	17,70	0,56	0,51	0,59	0,55
P131	42	male	t5	N	Plurihormonal_PIT-1	18,60				
P133	55	female	t6A	N	Somatotroph	17,86				

Supplemental Table S3. Biological characteristics of the 82 tumors from cohort II

	Onco-gene gsp	Methylation level of A/B DMR (%)	GNAS 1000*copies/ copies of BGUS	SSTR2 1000* copies/ copies of BGUS	AIP 100* copies/ copies of BGUS	GNAS allelic quantification (%)
1	gsp+	37	461,81	825,74		
2	gsp+	47	593,99	741,78	3127,8	24
3	gsp+		292,91	85,1	1069,44	9,95
4	gsp+	36	146,28	1885,3		
5	gsp+	40	330,31	253,31	1048,94	
6	gsp+	46	16,36	24,66	324,2	
7	gsp+	39	174,88	22,09	691,24	26,9
8	gsp+	42				
9	gsp+	30	728,82	65,94	1268,37	7,8
10	gsp+	38	332,3	85,6	1777,67	17,45
11	gsp+	40	576,55	46,5	983,12	
12	gsp+	41	206,93	335,02	1005,2	
13	gsp+	41				
14	gsp+	50	475,34	106,43	1730,31	20,5
15	gsp+	51	107,77	115,27	609,54	
16	gsp+	52	376,07	126,2	1437,47	
17	gsp+	42	239,49	771,07		4,5
18	gsp+	43	272,57	20,89		
19	gsp+	48	27,35	43,95	179,1	
20	gsp-	25	288,24	242,82	575,67	
21	gsp-	28	173,88	7,71	382,31	25,5
22	gsp-	33	466,55	131,71		
23	gsp-	36	841,17	23,95	4569,59	
24	gsp-	37	898,08	65,13	121,3	28,95
25	gsp-	37	323,87	378,46	1233,09	
26	gsp-	38	633,13	188,41	2011,05	17,6
27	gsp-	38	56,75	349,59	448,68	
28	gsp-	38	667,93	209,03		35,5
29	gsp-	38	167,75	12,24	1494,42	47,35
30	gsp-	38	206,08	508,01		
31	gsp-	39	755,12	1045,47	2074,66	2,35
32	gsp-	39	170,44	135,52	565,03	21,6
33	gsp-	39	935,66	89,26	1794,53	

34	gsp-	39	631,45	155,7	890,33	38,85
35	gsp-	39	562,13	1359,01		
36	gsp-	39				
37	gsp-	39	741,03	242,61	2952,41	21,6
38	gsp-	40	1063	197,09	2022,09	36,8
39	gsp-	41	82,77	1262,43		14,7
40	gsp-	41	1132,03	197,21	2429,13	7,65
41	gsp-	41	392,59	238,62	1363,08	
42	gsp-	42	178,56	33,43	171,47	
43	gsp-	42	1226,71	153,59	3752,35	5,25
44	gsp-	42	998,68	69,18	2415,22	14,65
45	gsp-	42	235,59	146,93		
46	gsp-	42	49,39	2,57	99,4	
47	gsp-	43	81,55	85,07	1211,18	29,1
48	gsp-	43	734,4	131,72	2671,26	
49	gsp-	43	1021,4	32,84	1636,07	8,05
50	gsp-	43	310,93	72,59	507,07	20,35
51	gsp-	43	141,16	329,24	1348,92	13,2
52	gsp-	44	603,43	257,05	1890,23	47,85
53	gsp-	45	1593,18	1125,84	7171,48	
54	gsp-	46	997,19	59,43	1639,18	
55	gsp-	47	404,7	497,6	2806,94	12,85
56	gsp-	48	603,61	7,72	3907,09	
57	gsp-	48	885,2	27,08	5304,56	
58	gsp-	48	692,45	141,89	1330,69	0,25
59	gsp-	49	684,49	770,12	1987,46	5,1
60	gsp-	49	1359,39	3822,41		
61	gsp-	50	556,9	162,66	778,88	
62	gsp-	50	857,55	400,14	3373,65	18,05
63	gsp-	50	474,33	1122,09		3,85
64	gsp-	50	195,44	9,18	332,48	42,6
65	gsp-	50	521,35	267,75	2079,68	
66	gsp-	51	392,8	171,9	3789,69	9,7
67	gsp-	51,8	247			
68	gsp-	52				14,5
69	gsp-	52				32,05
70	gsp-	53	1699,2	234,72	1672,37	8,8
71	gsp-	55	333,88	70,62	1906,2	

72	gsp-	56	41,64	3,75	567,34	
73	gsp-	56	897,7	82,07	2719,93	
74	gsp-	59	283,95	81,61	1315,53	
75	gsp-	60	773,52	138,95	1699,91	7,05
76	gsp-	60	427,48	45,52	1072,73	
77	gsp-	60,7	1116			
78	gsp-	62	870,94	270,09	2633,94	16,2
79	gsp-	62	92			
80	gsp-	69	28,43	8,48	307,62	
81	gsp-	81	17,54	1,8	82,44	
82	gsp-		404,7	99,16	5157,95	23,5
